# Prenatal risk factors and neonatal DNA methylation in very preterm infants

**DOI:** 10.1186/s13148-021-01164-9

**Published:** 2021-09-10

**Authors:** Marie Camerota, Stefan Graw, Todd M. Everson, Elisabeth C. McGowan, Julie A. Hofheimer, T. Michael O’Shea, Brian S. Carter, Jennifer B. Helderman, Jennifer Check, Charles R. Neal, Steven L. Pastyrnak, Lynne M. Smith, Lynne M. Dansereau, Sheri A. DellaGrotta, Carmen J. Marsit, Barry M. Lester

**Affiliations:** 1 Department of Psychiatry and Human Behavior, Alpert Medical School of Brown University, Providence, RI USA; 2 Department of Pediatrics, Women and Infants Hospital of Rhode Island, 101 Dudley Street, Providence, RI 02905 USA; 3 Gangarosa Department of Environmental Health, Emory University Rollins School of Public Health, Atlanta, GA USA; 4 Department of Epidemiology, Emory University Rollins School of Public Health, Atlanta, GA USA; 5 Department of Pediatrics, Alpert Medical School of Brown University, Providence, RI USA; 6 Department of Pediatrics, University of North Carolina at Chapel Hill School of Medicine, Chapel Hill, NC USA; 7 Department of Pediatrics-Neonatology, Children’s Mercy Hospital, Kansas City, MO USA; 8 Department of Pediatrics, Wake Forest School of Medicine, Winston-Salem, NC USA; 9 Department of Pediatrics, University of Hawaii John A. Burns School of Medicine, Honolulu, HI USA; 10 Department of Pediatrics, Spectrum Health-Helen DeVos Hospital, Grand Rapids, MI USA; 11 Department of Pediatrics, Harbor-UCLA Medical Center, Torrance, CA USA

**Keywords:** Prenatal, Methylation, Epigenetics, Epigenome-wide association study (EWAS), Neonatal, Preterm, Buccal

## Abstract

**Background:**

Prenatal risk factors are related to poor health and developmental outcomes for infants, potentially via epigenetic mechanisms. We tested associations between person-centered prenatal risk profiles, cumulative prenatal risk models, and epigenome-wide DNA methylation (DNAm) in very preterm neonates.

**Methods:**

We studied 542 infants from a multi-center study of infants born < 30 weeks postmenstrual age. We assessed 24 prenatal risk factors via maternal report and medical record review. Latent class analysis was used to define prenatal risk profiles. DNAm was quantified from neonatal buccal cells using the Illumina MethylationEPIC Beadarray.

**Results:**

We identified three latent profiles of women: a group with few risk factors (61%) and groups with elevated physical (26%) and psychological (13%) risk factors. Neonates born to women in higher risk subgroups had differential DNAm at 2 CpG sites. Higher cumulative prenatal risk was associated with methylation at 15 CpG sites, 12 of which were located in genes previously linked to physical and mental health and neurodevelopment.

**Conclusion:**

We observed associations between prenatal risk factors and DNAm in very preterm infants using both person-centered and cumulative risk approaches. Epigenetics offers a potential biological indicator of prenatal risk exposure.

**Supplementary Information:**

The online version contains supplementary material available at 10.1186/s13148-021-01164-9.

## Background

Infants born less than 30 weeks postmenstrual age (PMA) are at increased risk for adverse health and developmental outcomes. As children, they are at high risk for experiencing chronic health problems related to brain injury, including cerebral palsy, autism spectrum disorder, seizures, epilepsy, mental health disorders [1–4] and developmental delay in motor, language, and cognitive domains [1–3, 5–7]. However, there is also marked heterogeneity in outcomes [8–10]. For example, a recent follow-up study from the Neonatal Research Network (NRN) cohort of infants born extremely preterm found that by age 2, one quarter (24%) of children had no neurodevelopmental impairment, and 45% had only suspected or mild impairment [7].

Adverse prenatal conditions contribute to risk of preterm birth, and may also exacerbate the risk of negative outcomes associated with immaturity and illness in very preterm children [11]. For example, maternal mood disorders (e.g., depression, anxiety) and medical complications (e.g., pre-eclampsia, pre-pregnancy obesity) during pregnancy predict poorer neurobehavioral outcomes in very preterm neonates [11], which in turn are associated with longer term impairments [12]. Sociodemographic risk factors, such as low socioeconomic status, are also associated with poor developmental outcomes for very preterm children [13]. While these adverse conditions arise from unique sources (physical, psychological, and sociodemographic), they may impact similar biological systems and could have additive effects on the developing fetus. Therefore, exposure to a greater number or specific combinations of risk factors in the prenatal environment may contribute to the heterogeneous outcomes observed among preterm children.

One mechanism by which prenatal conditions may alter child neurodevelopment is via epigenetic processes [14]. Epigenetics refers to molecular processes that regulate gene expression without altering the underlying DNA sequence. DNA methylation (DNAm) is the most commonly studied epigenetic mechanism in humans and involves addition of a methyl group to a cytosine-phosphate-guanine (CpG) dinucleotide on a strand of DNA. DNAm plays an important role in regulating gene activity and expression. Additionally, offspring DNAm is sensitive to variations in environmental experience [15–17] and therefore may provide information about the biological embedding of prenatal conditions [14, 18–20].

Perhaps the most studied of all prenatal risk factors are those indicative of maternal psychological distress, including perceived stress and mood disorders [21]. These factors have also been studied in a growing number of candidate gene and epigenome-wide association studies (EWAS) [22, 23]. While candidate gene studies show associations between psychological risk factors in pregnancy and DNAm in genes implicated in offspring stress response systems [15], more recent EWAS on the same psychological risk factors have produced mixed findings [24–31], with differences not easily explained by study sample size. Therefore, the extent to which psychological risk factors in pregnancy impact offspring DNAm remains unclear. Physical risk factors (e.g., pre-pregnancy body mass index [BMI]) have also been investigated in relation to offspring DNAm and have similarly been associated with differential neonatal DNAm at a handful of CpGs [32]. These previous findings should be interpreted in light of several limitations, including the use of small sample sizes, exclusive use of cord blood for DNA sampling, and use of convenience or low-risk samples. Finally, most previous studies have investigated individual risk factors (e.g., depression, obesity) in isolation, rather than comprehensively assessing multiple facets of prenatal stress.

In this study, we investigated the relationship between prenatal risk factors and DNAm using buccal cell specimens in a high-risk population: children born very preterm. In addition, we studied prenatal risk comprehensively using two multiple-risk-factor approaches, rather than an individual variable approach. We first used cumulative risk models to investigate the additive burden of increasing *number* of risk factors on neonatal DNAm. Second, we used person-centered models to investigate the relationship of different *types* of risk factors with neonatal DNAm. Person-centered approaches such as latent class (LCA) and latent profile analysis (LPA) group individuals with similar co-occurring risk factors or phenotypes into mutually-exclusive groups. Whereas one previous study investigated cumulative prenatal risk in association with neonatal DNAm [24], person-centered models have not yet been used to study the association between prenatal risk phenotypes and neonatal DNAm. Therefore, the goals of this study were to examine relations among prenatal risk factors and DNAm in very preterm neonates and to understand whether these relations differ depending on whether cumulative risk or person-centered models are used. Addressing these goals may enable us to identify important biological mechanisms underlying the association between prenatal environmental experiences and child outcomes and will provide information regarding how best to operationalize prenatal risk factors in future studies of neonatal health.

## Methods

### Study population

The Neonatal Neurobehavior and Outcomes in Very Preterm Infants (NOVI) study enrolled infants born < 30 weeks postmenstrual age (PMA) from nine NICUs affiliated with six universities from April 2014 to June 2016. Inclusion criteria included: (a) birth < 30 weeks PMA; (b) parental ability to read and speak English or Spanish; and (c) residence within 3 h of the NICU and follow-up clinic. Infants were excluded for major congenital anomalies [33], NICU death, maternal age < 18 years, maternal cognitive impairment, or maternal death.

Parents of eligible infants were invited to participate in the study when survival to discharge was determined to be likely by the attending neonatologist. Study procedures were explained and informed consent was obtained in accordance with each institution’s review board. Children were included in this analysis if they were enrolled in NOVI at birth and had a neonatal buccal swab collected (*M*_PMA_ = 39.2 weeks). There were 704 infants enrolled in NOVI; of these 651 (92%) had parental consent to obtain buccal swabs. Mothers were interviewed at enrollment to obtain demographic information (age, education, occupation, race/ethnicity, and marital/cohabitation status). Information regarding prenatal substance use, physical health, and psychological health were obtained via maternal interview and medical record review.

### Measures

#### Prenatal risk factors

We assessed 24 prenatal risk factors in four domains: demographic (5 items), substance use (4 items), physical health (9 items), and psychological health (6 items). Individual risk factors were assessed via maternal interview and medical record review.

Demographic risk factors included maternal age > 35 years, low socioeconomic status (Hollingshead category 5) [34], maternal education less than a high school degree, minority race or ethnicity, and no romantic partner. Substance use items included maternal use of tobacco, alcohol, marijuana, or other illegal substances (e.g., heroin, cocaine) as noted in her medical record.

Physical health risks included maternal underweight (BMI < 18.5) and obesity (BMI ≥ 30), calculated from reported pre-pregnancy height and weight. Gestational weight gain that exceeded Institute of Medicine guidelines [35] was also determined using calculated BMI and reported weight gain. Maternal hypertension, pre-eclampsia, diabetes, HIV/AIDS or other sexually transmitted infection, any other infection, and receipt of prenatal care were all determined from medical record review.

Psychological health risks included maternal depression and anxiety and maternal moods and feelings. Maternal depression during pregnancy was determined from medical record or maternal report of anti-depressant use, or from maternal report of depression diagnosis, treatment, or counseling during pregnancy. The same method was used to determine maternal anxiety during pregnancy. Beyond diagnosed mental health disorders, maternal moods and feelings during pregnancy were assessed from four questions asking mothers to indicate the extent to which (a) their pregnancy was a hard time in their lives, and the extent to which they felt (b) down, (c) hopeless, and (d) slow during their pregnancy. Risk was determined by responses indicating that pregnancy was a “very hard time” or “one of the worst times” in their lives, or if mothers indicated they “often” or “always” felt down, hopeless, or slow [36].

#### Neonatal DNA methylation (DNAm)

Genomic DNA was extracted from buccal swab samples, collected near term-equivalent age, using the Isohelix Buccal Swab system (Boca Scientific), quantified using the Quibit Fluorometer (Thermo Fisher, Waltham, MA, USA) and aliquoted into a standardized concentration for subsequent analyses. DNA samples were plated randomly across 96-well plates and provided to the Emory University Integrated Genomics Core for bisulfite modification using the EZ DNA Methylation Kit (Zymo Research, Irvine, CA), and subsequent assessment of genome-wide DNAm using the Illumina MethylationEPIC Beadarray (Illumina, San Diego, CA) following standardized methods based on the manufacturer’s protocol.

Pre-processing of data followed a modified workflow described elsewhere [37]. Array data were normalized via Noob normalization [38, 39] and samples with more than 5% of probes yielding detection *p*-values > 1.0E-5 or mismatch between reported and predicted sex were excluded. In addition, one of two duplicated samples was omitted (retained duplicated sample with smallest detection *p*-values). Probes with median detection *p*-values < 0.05, probes measured on the X or Y chromosome, probes that had single nucleotide polymorphisms (SNP) within the binding region or that could cross-hybridize to other regions of the genome were excluded [40]. Then, array data were standardized across Type-I and Type-II probe designs with beta-mixture quantile normalization [41, 42]. After exclusions, 706,323 probes were available from 542 samples for this study (83% of 651 with buccal swab consent; 77% of entire NOVI cohort). These data are accessible through NCBI Gene Expression Omnibus (GEO) via accession series GSE128821.

#### Covariates

DNAm varies by cell type and cellular heterogeneity is a documented source of confounding in EWAS that make use of mixed cell samples [43]. A variety of cell-type deconvolution methods have been developed to estimate cell type proportions based on cell-type specific DNAm pattern. We estimated the proportion of epithelial, fibroblast, and immune cells (e.g., B-cells, natural killers, CD4 + and CD8 + T-cells, monocytes, neutrophils, eosinophils) in our buccal samples using reference methylomes [44]. As previously shown [45], for 95% of our buccal samples, 95.7% of the cells were epithelial cells, with the remainder being immune cells. Given the strong inverse correlation between epithelial and immune cell proportions, cellular heterogeneity was adjusted for by including the proportion of epithelial cells as a covariate in all statistical models.

In addition to cellular heterogeneity, our EWAS models controlled for child sex, recruitment site, and PMA at buccal swab. We accounted for potential batch effects by controlling for sample plate.

### Statistical analysis

#### Prenatal risk classes and index

We first conducted latent class analysis (LCA) to categorize subgroups of women with similar prenatal risk factors. LCA is a statistical method for classifying individuals into mutually exclusive groups, or latent classes, based on their pattern of responses to a set of categorical indicator variables. The method is considered latent because true group membership is unknown. LCA employs maximum likelihood estimation. The optimal number of latent classes is determined using fit statistics and interpretibility of the models. For these analyses, we fit LCA models to our 24 observed risk factors and examined solutions ranging from 1 to 4 classes. All models were run in Mplus 8.4. We used posterior probabilities from the best fitting LCA model to classify women into distinct subgroups. We describe the subgroups in terms of how they differ on the 24 prenatal risk factors.

Next, we created a cumulative prenatal risk index that measured the total number of risk factors experienced by mothers. One point was assigned for each of 24 risk factors and a proportion score was created by dividing this sum by the total number of items mothers responded to. A proportion was used rather than a sum because of the possibility of item nonresponse. However, the majority of mothers (96%) had data for all 24 items.

#### Epigenome-wide association study (EWAS)

All EWAS analyses were conducted in R 4.0.2. We used generalized estimating equations (GEE) that accounted for the nesting of children within families. GEE models were run on logit transformed ($\mathrm{log}\left(\frac{\beta }{1-\beta }\right)$) DNAm data that approximated a Gaussian distribution. For easier interpretation of significant CpG sites, we present model coefficients obtained from both transformed and untransformed (beta-values) data, where the latter can be interpreted in terms of percent methylation at a given CpG site. To account for multiple testing, *p*-values were adjusted using a Bonferroni correction (*α* = 0.05/706323). We conducted separate EWAS analyses with latent prenatal class (3-level factor) and cumulative prenatal risk (continuous) as the focal independent variables and compare our findings from the two types of models. We report results from models that yielded suggestive associations (FDR < 10%) in Additional file 1 and report those results that were significant with Bonferroni-correction (706,323 tests) herein.

One challenge of EWAS in humans is the inaccessibility of tissues of interest, namely brain tissue. Although we rely on peripheral tissues such as buccal cells, there is variability in the extent to which peripheral DNAm is associated with DNAm in the brain. For CpGs that were significantly associated with prenatal risk in either latent class or cumulative risk models, we examined the correlation between DNAm in buccal and brain tissue [46]. This additional information can help us determine which of our significant CpGs may have similar patterns of DNAm in buccal and brain tissue.

To determine the biological functions of CpGs associated with prenatal risk, we conducted gene enrichment analyses using the *gometh* function in the *MissMethyl* package [47]. This procedure accounts for the number of CpGs annotated to each gene. We examined both pathway-based gene sets (i.e., KEGG and gene ontology (GO) terms). For enrichment analyses, we included CpGs that were associated with prenatal risk at an FDR of < 5%. Overrepresentation results within a 10% FDR were deemed statistically significant. We also aimed to identify whether any CpGs associated with prenatal risk were within genes that have been linked with neurodevelopmental phenotypes. Thus, based on the genes that were annotated to our significant CpGs, we additionally annotated these CpGs with traits that have been linked to these genes via prior genome-wide association studies (GWAS) using the NHGRI-EBI GWAS catalog [48].

All analyses described thus far have described methods for estimating the association between prenatal risk and individual CpGs. However, DNAm is generally highly correlated at adjacent CpG sites [49]. To better understand whether our EWAS results are limited to individually significant CpGs or are more broadly representative of regions of the genome that are differentially methylated, we additionally conducted differentially methylated regions (DMR) analysis using the *dmrff* package [50].

## Results

### Study population

The NOVI study included 704 infants born to 601 mothers. All mothers were included in the LCA analysis. Of the 651 potential buccal swabs, 624 (96%) were collected. Missing data were due to technical sampling or handling error, missing swabs, or unscheduled discharges prior to swabs being obtained. Of the 624 infants with buccal swabs, there were 542 infants (from 470 mothers) with DNAm data that passed quality control steps (described earlier).

Maternal and child characteristics are summarized in Table 1. Those without DNAm data were more likely to be low SES (*p* = 0.04) and to be a minority race or ethnicity (*p* = 0.004), compared to those with DNAm data. Included and excluded children did not differ based on prenatal risk class or cumulative prenatal risk (all *p* > 0.05).

**Table 1:** Demographic and medical characteristics of the sample

Sample characteristics	Full sample (*N* = 601)	Included (*N* = 470)	Excluded (*N* = 131)	*p*-value
Maternal characteristics*				
Maternal education: < HS/GED	13% (79/598)	15% (68/468)	8.5% (11/130)	0.07
Low SES: Hollingshead = 5	9.9% (59/599)	8.5% (40/469)	15% (19/130)	0.04
Minority race or ethnicity^±^	58% (347/601)	55% (257/470)	69% (90/131)	0.004
No partner	25% (152/600)	26% (124/470)	22% (28/130)	0.26

Neonatal characteristics	Full sample (*N* = 704)	Included (*N* = 542)	Excluded (*N* = 162)	*p*-value
Infant gender = Male	56% (388/697)	55% (299/539)	56% (89/158)	0.85
Multiple gestation	26% (184/697)	27% (145/539)	25% (39/158)	0.58
Cesarean delivery	71% (495/696)	71% (382/539)	72% (113/157)	0.79
PMA at Birth (weeks)	27.0 ± 1.92	27.0 ± 1.92	27.0 ± 1.92	0.86
Birth weight (grams)	948.3 ± 280.6	951.1 ± 281.8	938.5 ± 277.0	0.62
Head circumference (cm)	24.5 ± 2.43	24.5 ± 2.48	24.4 ± 2.24	0.70
PMA at Discharge (weeks)	40.5 ± 5.43	40.3 ± 5.20	41.29 ± 6.12	0.05
Length of NICU stay (LOS days)	93.5 ± 41.9	91.7 ± 40.1	99.7 ± 47.4	0.05
Weight at discharge (grams)	3013 ± 905	3001 ± 861	3057 ± 1042	0.50
Severe retinopathy of prematurity	5.9% (41/697)	6.3% (34/539)	4.4% (7/158)	0.38
Necrotizing enterocolitis/sepsis	18% (128/697)	19% (103/539)	16% (25/158)	0.35
Chronic lung disease	51% (357/697)	51% (277/539)	51% (80/158)	0.87
Serious brain injury^+^	13% (92/694)	13% (69/539)	15% (23/155)	0.51

Means ± standard deviations (continuous) or percentage and frequencies (categorical) of demographic and medical characteristics. *p*-values refer to the comparison of included versus excluded individuals and were obtained from t-tests (continuous variables) and chi-squared tests (categorical variables)

PMA, postmenstrual age; HS, high school; GED, General Equivalency Diploma; SES, socioeconomic status

*All mothers with prenatal data were included in the latent class analysis. Included versus excluded in this Table refers to individuals with data for the epigenome-wide analysis

^±^Minority race or ethnicity was defined as any non-White race (e.g., Black, Asian) or ethnicity (e.g., Hispanic and/or Latino/a)

^+^Serious brain injury included parenchymal echodensity, periventricular leukomalacia, or ventricular dilation diagnosed via cranial ultrasound

### Prenatal risk

We first estimated LCA models and used standard model fit statistics to determine the ideal number of latent profiles. Lo-Mendell-Rubin and bootstrapped loglikelihood ratio tests indicated that the 4-profile model did not fit significantly better than the 3-profile model, but that the 3-profile model did fit significantly better than the 2-profile model. The 3-profile solution had the lowest Bayesian information criterion (BIC), high entropy (0.84), and high class probabilities (0.89–0.94). The class sizes were reasonable (smallest class = 13%) and the classes were readily interpretable. Thus, model fit statistics supported a 3-profile LCA solution. Fit statistics for all LCA solutions are included in Additional file 2.

**Fig. 1:**
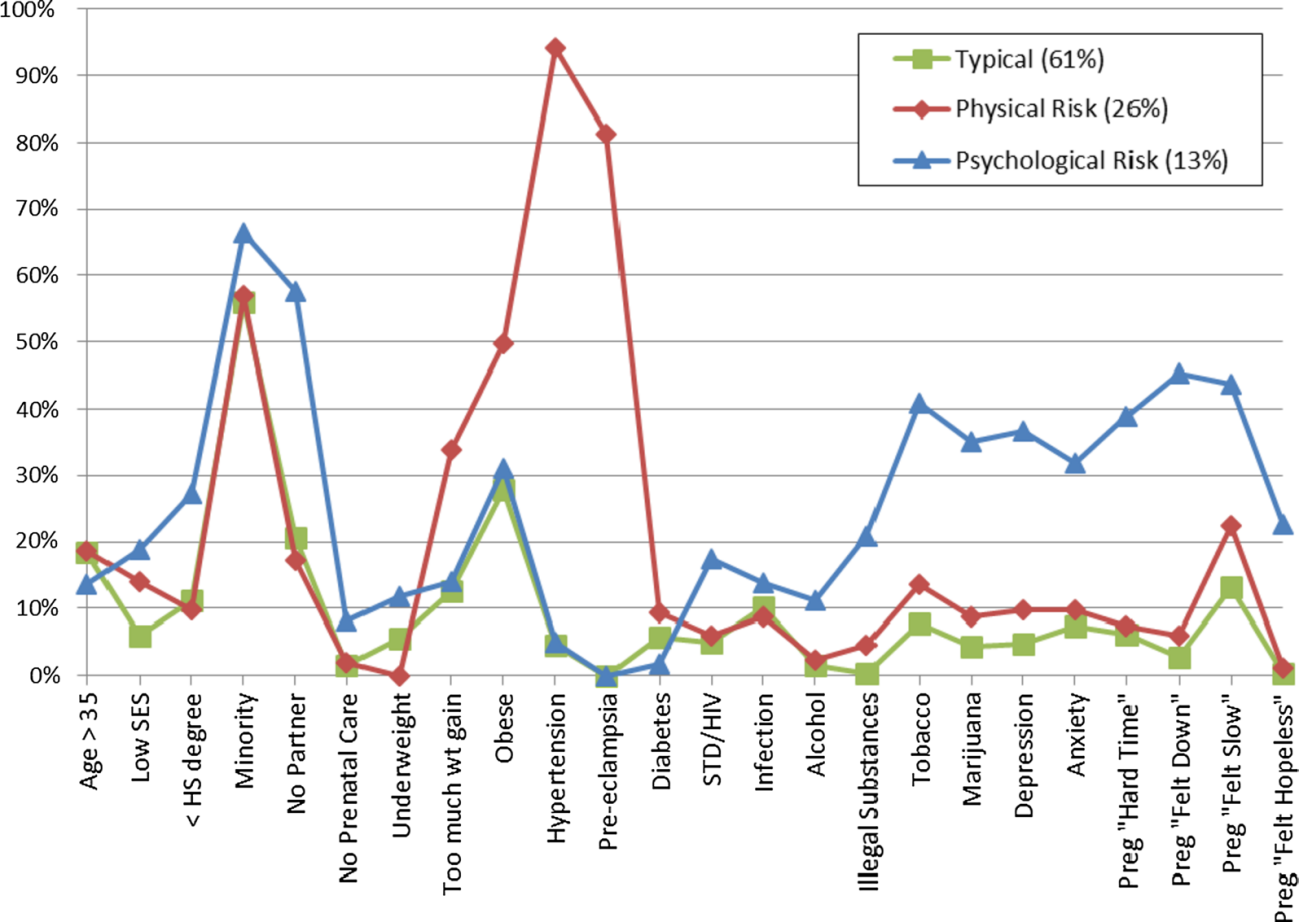
Rates of endorsement of 24 prenatal risk factors by latent class membership. Women in class 1 (green; 61%) endorse few prenatal risk factors. Women in class 2 (red; 26%) endorse elevated physical health problems, whereas women in class 3 (blue; 13%) endorse elevated substance use and psychological problems

Women in the three latent classes differed on 21 out of 24 prenatal risk factors (Table 2). Figure 1 depicts differences in the 3 classes by rates of endorsement of prenatal risk factors. Women in class 1 (“Typical”; 61%) had the lowest rates of endorsement for all risk factors. In contrast, women in class 2 (“Physical Risk”; 26%) exhibited elevated physical health problems, including high rates of obesity, hypertension, and pre-eclampsia. Women in class 3 (“Psychological Risk”; 13%) exhibited elevated substance use and psychological health problems. They endorsed high rates of alcohol, tobacco, and drug use during pregnancy, as well as high rates of anxiety and depression. Women in class 3 were also more likely to indicate that they felt “down”, “slow”, and “hopeless” during their pregnancy, and to indicate that their pregnancy was a “very hard time” in their lives.

**Table 2:** Distribution of individual prenatal risk factors in full sample and by latent class

Prenatal risk factors	Full sample (*N* = 601)	Typical (*N* = 367; 61%)	Physical risk (*N* = 155; 26%)	Psychological risk (*N* = 79; 13%)	*χ*^2^
Age > 35	18%	18%	18%	14%	0.89
Low SES	10%	6.5%	14%	18%	12.78**
< HS degree	13%	12%	9.8%	26%	12.54**
Minority	58%	57%	56%	66%	2.45
No partner	25%	21%	18%	62%	65.33***
No prenatal care	2.7%	1.6%	1.9%	9.1%	14.01***
Underweight	5.0%	5.8%	0.0%	12%	15.48***
Obese	34%	26%	53%	33%	35.11***
Too much weight gained	18%	12%	34%	17%	33.93***
Hypertension	27%	3.3%	95%	3.9%	490.33***
Pre-eclampsia	21%	0.0%	79%	0.0%	442.57***
Diabetes	6.0%	5.5%	9.7%	1.3%	7.00*
STI/HIV	7.0%	5.0%	6.5%	18%	16.33***
Infection	10%	10%	9.0%	14%	1.39
Alcohol	3.2%	1.4%	2.6%	13%	27.18***
Illegal substances	4.5%	0.3%	4.5%	24%	85.30***
Tobacco	14%	8.0%	14%	44%	70.36***
Marijuana	10%	4.1%	8.4%	41%	96.07***
Depression	11%	4.9%	10%	39%	79.51***
Anxiety	12%	7.4%	9.7%	35%	50.62***
Pregnancy “Hard Time”	11%	6.7%	6.5%	42%	86.18***
Pregnancy “Felt Down”	9.9%	2.7%	5.9%	51%	173.87***
Pregnancy “Felt Slow”	20%	13%	23%	46%	44.90***
Pregnancy “Felt Hopeless”	3.9%	0.3%	1.3%	26%	115.30***

STI, sexually transmitted infection; HIV, Human immunodeficiency virus

*p < .05, **p < .01, ***p < .001

We then calculated a cumulative prenatal risk score for each participant. On average, mothers experienced an average of 3.6 risk factors (*SD* = 2.3), with a range from 0 to 12.

### Epigenome-wide association study with prenatal risk profiles

Our first set of models compared DNAm for children born to women in the Physical Risk or Psychological Risk groups to children born to women in the Typical group. Results are displayed in Table 3. After Bonferroni adjustment, one CpG was differentially methylated in the Physical Risk group and one CpG was differentially methylated in the Psychological Risk group. Compared to the Typical group, neonates of mothers in the Physical Risk group had, on average, 5% lower DNAm at the identified CpG (cg25123362) located in the body of the *BNIP3* gene. Neonates of mothers in the Psychological Risk had 4% lower DNAm at the identified CpG (cg08930413) located in the body of the *PRKAG2* gene.

**Table 3:** Epigenome-wide association study results for CpG sites that yielded significant associations after Bonferroni adjustment

CpG	Location	Gene annotation	Coefficient (m-value)	*p* value (Raw)	*p* value (Adj)	Coefficient^a^ (beta-value)	Brain-buccal correlation
*Model 1: Physical Risk vs. Typical*							
cg25123362	Chr10: 133793734	*BNIP3* (Body)	− 0.25	2.76E−11	1.95E−05	− 0.05	− 0.09
*Model 1: Psychological Risk vs. Typical*							
cg08930413	Chr7: 151548036	*PRKAG2* (Body)	− 0.28	2.11E−08	1.49E−02	− 0.04	0.22
*Model 2: Cumulative Prenatal Risk*							
cg16999677	Chr5: 843982	*ZDHHC11* (Body)	− 2.05	6.85E−08	4.84E−02	− 0.05	0.61**
cg05324191	Chr1: 116994757	LOC101929023 (Body)	1.03	1.30E−09	9.18E−04	0.02	− 0.33
cg01533736	Chr20: 22542854	*LINC00261* (Body)	− 1.18	1.17E−09	8.25E−04	− 0.03	0.40
cg00569188	Chr21: 41122530	*IGSF5* (Body)	− 1.28	4.61E−08	3.26E−02	− 0.03	− 0.17
cg09979763	Chr1: 245499904	*KIF26B* (Body)	− 2.02	4.23E−08	2.99E−02	− 0.03	− 0.07
cg11420269	Chr16: 70516713	*COG4* (Body; ExonBnd)	− 1.46	2.07E−08	1.46E−02	− 0.03	0.02
cg27514986	Chr15: 39486981	*C15orf54*^+^	2.78	9.74E−09	6.88E−03	0.05	0.19
cg05636131	Chr7: 148844053	*ZNF398* (5’ UTR; TSS1500)	− 1.09	6.93E−08	4.90E−02	− 0.02	0.42^‡^
cg11531492	Chr3: 125673505	*ROPN1B*^+^	− 2.63	6.56E−08	4.63E−02	− 0.05	− 0.08
cg26760502	Chr14: 105493800	*CDCA4*^+^	− 0.88	4.89E−08	3.45E−02	− 0.01	− 0.09
cg19573457	Chr22: 25893657	*CRYBB2P1*^+^	− 1.68	2.17E−08	1.53E−02	− 0.04	0.44*
cg01284858	Chr10: 123902371	*TACC2* (5’UTR; Body)	3.61	6.20E−09	4.38E−03	0.06	− 0.32
cg12155575	Chr3: 186965150	*MASP1* (Body)	− 1.16	8.97E−09	6.33E−03	− 0.03	0.08
cg11221492	Chr14: 36790270	*MBIP* (TSS1500)	− 0.90	3.92E−08	2.77E−02	− 0.01	0.54*
cg22102865	Chr7: 148844067	*ZNF398* (TSS1500; 5’ UTR)	− 1.24	1.37E−08	9.64E−03	− 0.02	0.46*

^a^Note that the coefficient for cumulative risk models represents the expected increase in % DNAm associated with a 10% increase in risk. ^‡^*p* < .10, *p < .05, **p < .01, ^+^ indicates closest gene. Adjusted *p*-value is Bonferroni corrected

### Epigenome-wide association study with cumulative prenatal risk

**Fig. 2:**
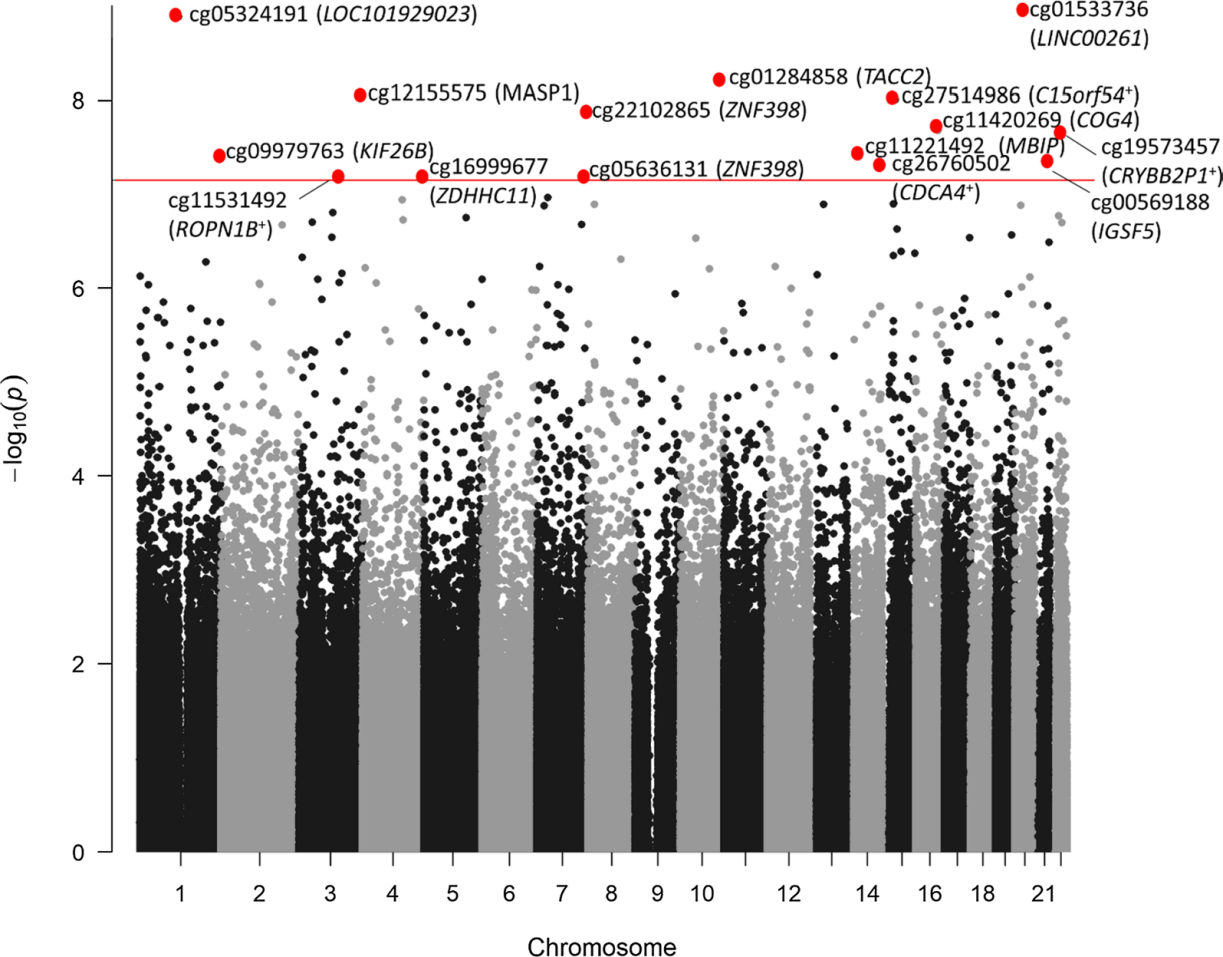
Manhattan plot of epigenetic loci associated with cumulative prenatal risk. The x-axis shows the genomic location of individual CpG sites and the y-axis shows the −log_10_(*p* values) from models relating cumulative prenatal risk to CpG methylation, adjusting for child sex, recruitment site, postmenstrual age at collection, sample batch, and cellular heterogeneity. Gene annotations have been added for all CpGs yielding significant associations after Bonferroni adjustment. The horizontal red line depicts the Bonferroni adjusted *p*-value threshold (*α* = 0.05/706323). ^+^indicates closest gene

Next, we tested for associations between cumulative prenatal risk and neonatal DNAm. We found 15 statistically significant CpGs that were differentially methylated with increasing cumulative prenatal risk (Fig. 2). Increasing prenatal risk was associated with lower DNAm at 12 CpGs and higher DNAm at 3 CpGs. These differences were small in magnitude and ranged from a 1–6% difference in DNAm associated with a 10% increase in cumulative prenatal risk.

### Brain-buccal correlations

We used the publicly available IMAGE-CpG website (http://han-lab.org/methylation/default/imageCpG) to explore whether DNAm levels in buccal tissue was correlated with DNAm levels in brain tissue [46], for the CpGs that were identified as significant in our EWAS models. While neither of the CpGs identified in our prenatal risk profile EWAS exhibited significant brain-buccal correlations, 4 of the 15 CpGs that were identified in our models of cumulative prenatal risk did exhibit significant brain-buccal correlations (*r* = 0.44 to 0.61, *p* < 0.05).

### Functional and phenotypic enrichment

Because few CpGs were significant in our prenatal risk profile EWAS, we considered in our enrichment analyses those CpGs that were significant within a 5% FDR in the cumulative prenatal risk model (*n* = 384 CpGs). After FDR correction, there were no significantly enriched pathway or gene ontology terms.

### CpG annotation

We identified phenotypes and traits that have been associated with the genes annotated to significant CpGs in our EWAS models (Table 4). Of the 17 significant CpGs, 9 were located in genes associated with neurobehavioral traits including cognitive ability, memory, reaction time, brain volume, and mental health disorders (e.g., depression, bipolar disorder, schizophrenia). Of these 9 CpGs within neurobehaviorally-linked genes, 3 had significant blood-buccal correlations (cg19573457 [*CRYBB2P1],* cg11221492 [*MBIP],* cg22102865 [*ZNF398*]).

**Table 4:** CpGs associated with prenatal risk are linked to genes that have been associated with traits in the GWASdb

Gene	Traits (N)	Selected traits
*BNIP3*	8	Self-reported educational attainment, intelligence, mathematical ability, household income, schizophrenia, cognitive function
*PRKAG2*	38	Self-reported educational attainment, mathematical ability, brain/neuroimaging measurement, gut microbiome measurement, white matter microstructure, cardiovascular disease, brain volume measurement, bipolar disorder, psychotic symptoms, cognitive function
*ZDHHC11*	1	Myopia age of onset
*LOC101929023*	–	–
*LINC00261*	4	Body mass index, fasting blood glucose measurement, birth weight
*IGSF5*	4	Systolic blood pressure, hypertension, short-term memory, health literacy
*KIF26B*	15	Brain volume/neuroimaging measurement, response to SSRI, unipolar depression, diet measurement, brain measurement, schizophrenia
*COG4*	3	Body height, body weight, body mass index
*C15orf54*	1	Dihydroxy docosatrienoic acid measurement
*ZNF398*	10	Brain volume/neuroimaging measurement, white blood cell count
*ROPN1B*	–	–
*CDCA4*	1	Telomere length
*CRYBB2P1*	4	Bipolar disorder
*TACC2*	16	Opioid dependence, metabolite measurement, body weight gain, schizophrenia, body height, reaction time measurement
*MASP1*	3	Type 2 diabetes
*MBIP*	6	Self-reported educational attainment, hypertension

Additionally, 11 of the 17 CpGs were located in genes associated with physical health markers, including body mass index, cardiovascular disease, hypertension, type-2 diabetes, and white blood cell counts. Of these 11 CpGs within health-linked genes, 3 had significant blood-buccal correlations (cg16999677 [*ZDHHC11*], cg11221492 [*MBIP],* cg22102865 [*ZNF398*]).

### DMR analysis

Last, we performed DMR analyses to test whether there were regional, not just CpG site-specific, differences in DNAm associated with prenatal risk factors. DMR analyses comparing the Physical Risk group to the Typical group found one significant region (Chr10: 133793734–133794558) containing three CpG sites (cg25123362, cg12751948, cg16592121). Children born to mothers in the Physical Risk group had less methylation in this region, on average, compared to children born to mothers in the Typical group (*p* = 4.17E-11). This region contained the individually significant CpG described above in the Physical Risk versus Typical model (cg25123362) and this DMR similarly annotated to the *BNIP3* gene.

Analyses comparing the Psychological Risk group to the Typical group also resulted in one significant DMR (Chr14: 77785784–77785968) containing two CpG sites (cg02181287, cg03738767). Children born to mothers in the Psychological Risk group had less methylation in this region, on average, compared to children born to mothers in the Typical group (*p* = 4.65E-08). This DMR was in a different genomic location compared to the individually significant CpG described earlier and annotated to the *GSTZ1* and *POMT2* genes.

Finally, we found six DMRs that were significantly associated with cumulative prenatal risk. Five of the six were negatively associated with prenatal risk, suggesting lower DNAm with increasing levels of risk. One DMR was positively associated, suggesting more DNAm with increasing levels of risk. One of the six significant DMRs (Chr7: 148843026–148844053) was in a similar region as a CpG (cg22102865) that was identified as being individually significant. One region (Chr14: 77785784–77785968) that was identified as a DMR related to cumulative prenatal risk was also identified as a significant DMR in the comparison of the Psychological Risk group to the Typical group. The other four DMRs (Chr3: 186965021–186965150; Chr1: 155659719–155659882; Chr22: 25884154–25884537; Chr15: 34260712–34260956) did not share CpGs in common with other DMR analyses or with the individual CpG results. These four DMRs annotated to the following genes: *MASP1*, *DAP3*, *YY1AP1*, *CRYBB2P1*, *AVEN*, and *CHRM5*. Full results for the DMR analysis are included in Additional file 3.

## Discussion

We conducted an epigenome-wide study to test the associations between prenatal risk factors and neonatal DNAm in a sample of very preterm neonates. LCA findings showed 3 distinct prenatal risk groups; a group with few risk factors (“Typical”; 61%) and groups with elevated physical (26%) or psychological (13%) risk factors. Neonates born to women in these higher risk subgroups had differential DNAm patterns at two CpG sites. The cumulative prenatal risk analysis showed that a higher risk score was associated with greater methylation at 3 CpG sites and lower DNAm at 12 CpG sites.

The investigation of both the total number (cumulative score) and co-occurring types (LCA profiles) of prenatal risk factors as they relate to epigenome-wide DNAm in infants, including preterm infants, is novel. Previous EWAS have relied on a single variable approach with mixed findings. Two studies found no associations between psychological risk factors and neonatal DNAm [24, 25], several studies uncovered only a small number of differentially methylated CpGs [26–31], and one study found 145 differentially methylated CpGs [51]. The only other study using a cumulative risk approach found no significant associations between cumulative prenatal stress and neonatal DNAm [24]. However, this study measured DNAm from blood, used a different DNAm bead chip, included term as well as preterm children, and did not assess physical health risks. In contrast, the cumulative prenatal risk index used in the current study accounted for 24 demographic, substance use, physical health, and psychological health indicators. There was no overlap between significant CpGs or genes identified in this study and those identified in any of the previous EWAS on prenatal risk factors. These differences may be due to differences in methods (e.g., which risk factors were assessed; single versus multiple risk approach) and samples (e.g., primarily term versus exclusively preterm children).

We also investigated the relationship between prenatal stress phenotypes and neonatal DNAm by using LCA to classify women into subgroups on the basis of the same 24 risk factors. We found evidence for three distinct prenatal stress phenotypes. The majority of women belonged to a group that experienced few risk factors. In comparison, women in the physical risk group had elevated levels of medical risk factors, such as hypertension (95%) and pre-eclampsia (79%). Women in the psychological risk group had elevated levels of mental health concerns, including the highest rates of depression (39%) and anxiety (35%), as well as the highest rates of tobacco (44%), marijuana (41%), alcohol (13%), and illegal drug (24%) use. Previous work investigating prenatal stress phenotypes in relation to fetal and neonatal behavior [52] also found a group with elevated physical risk factors (e.g., higher blood pressure, greater calorie, fat, and sugar consumption) and a group with elevated psychological risk factors (e.g., greater depression, anxiety, and perceived stress) with similar proportions of women falling into the low risk or typical group (approximately 2/3) versus one of the two higher risk groups (approximately 1/3). These similarities emerged despite different study methodologies (e.g., maternal self-reported versus objectively assessed risk factors) and different variables included in the latent models. Taken together, these findings provide evidence for distinct subgroups of women who may differentially be impacted by physical health issues or psychological health issues during pregnancy. However, our study is the first to demonstrate associations between prenatal risk phenotypes and neonatal DNAm.

Among the 15 CpGs that were associated with cumulative prenatal risk, 12 were located in genes that have been linked in GWAS studies to relevant phenotypes for both physical (e.g., blood pressure [53], BMI [54], diabetes [55]) and mental health outcomes (e.g., schizophrenia [56], depression [57], bipolar disorder [58]) as well as neurodevelopmental markers (e.g., brain volume/measurement [59], reaction time [60]). One gene (*CDCA4*) identified in our analysis encodes a member of the E2F family of transcription factors, which regulate spindle organization, cytokinesis, and cell proliferation [61]. This gene has also previously been shown to be associated with leukocyte telomere length [62], suggesting potential ties between prenatal risk and biological aging processes. There was also some overlap in genes (*KIF26B*; *TACC2*) identified in the current analysis with genes we previously reported to be associated (FDR < 0.10) with atypical neurodevelopmental profiles in the same sample [45]. Therefore, it is possible that DNAm of these genes may play a role in explaining the prenatal programming of child neurodevelopment, although additional longitudinal studies would be needed to rigorously test this hypothesis.

Only 2 CpG sites were differentially methylated across prenatal risk groups. Neonates of mothers in the physical risk group had 5% less methylation, on average, at cg25123362, located in the *BNIP3* gene. Neonates of mothers in the psychological risk group had 4% less methylation, on average, at cg08930413, located in the *PRKAG2* gene. Interestingly, both the *BNIP3* and *PRKAG2* genes have been associated with similar traits in previous GWAS analyses, including educational attainment [63] and cognitive function [60, 63], suggesting that the associations between prenatal risk factors and child outcomes may be marked by some degree of equifinality (i.e., different biological pathways leading to the same outcome) [64].

Our DMR analyses yielded overlapping results with the individual CpG analyses for *ZNF398* (associated with cumulative risk) and *BNIP3* (associated with physical risk group). We also identified four significant DMRs that did not include individually significant CpGs from our EWAS, annotated to *MASP1*, *DAP3*, *YY1AP1*, *CRYBB2P1*, *AVEN*, and *CHRM5*. *ZNF398* and *BNIP3* are particularly interesting given that they were identified in both CpG-specific and regional analyses. Prior GWAS have linked *ZNF398* to brain volume and neuroimaging measurements [65], while *BNIP3* has been linked to cognitive function [60]. Additionally, we found that DNAm levels at one CpG within the *ZNF398* gene (cg22102865) were positively correlated between brain and buccal tissues from publicly available data [46].

It was notable that our analysis using cumulative prenatal risk identified more significant CpGs (*N* = 15) than our analyses investigating phenotypes of prenatal risk (*N* = 2). There was also no overlap in significant CpGs or genes identified by the two models, suggesting that they may be unique methods for identifying risk. Cumulative risk models are attractive because of their simplicity, parsimony, and relatively greater statistical power, compared to alternative approaches (e.g., individual risk variables) [66]. They also mimic how these factors impact pregnant women as they rarely occur in isolation. An empirical comparison of cumulative risk indices to either individual variable or factor score approaches also found that risk indices provide better prediction of developmental patterns [67]. Therefore, cumulative prenatal risk indices may be a useful approach in that they provide a strong signal for the relationship of early adversity to child outcomes, including DNAm. Indeed, there is precedence in the epigenetic literature for the use of cumulative risk scores as predictors of children’s DNAm [24, 68]. The disadvantages of cumulative risk indices are that each individual risk factor carries equal weight. Moreover, we cannot determine which variables are the most important drivers in explaining the association with outcomes which limits the practical application of these risk indices in clinical practice. Alternative “person-centered” approaches, like LCA and LPA models, allow for the modeling of patterns of correlated risk factors as they co-occur in real participants. An underlying assumption is that different types of risk are differentially associated with outcomes. “Person-centered” approaches offer advantages over individual variable or cumulative risk approaches in that they are both comprehensive *and* specific. The differentiation of prenatal risk phenotypes into physical and psychologically stressed individuals offer a new way to think about types of adverse prenatal environments that may be differentially related to child outcomes [52] and may require different types of intervention. Previous studies comparing cumulative risk and LPA approaches in the context of child development have similarly reported that these two types of analyses provide complementary information about the relationships between risk factors and outcomes [69].

Limitations of this study are to be appreciated. First, we only considered binary risk factors as opposed to continuous indicators. This decision was partly necessitated by our creation of a cumulative risk index. Many of the risk factors we included (e.g., presence or absence of physical or mental health diagnosis) are dichotomous in nature but some loss of information may have occurred from dichotomizing other variables (e.g., SES). Second, as the inclusion criteria for this study included birth prior to 30 weeks gestation and likely survival to discharge, women were necessarily recruited after pregnancy, and some pregnancy data were assessed retrospectively (e.g., maternal report of pregnancy moods and feelings). However, any retrospective data were collected in the neonatal period, potentially reducing the impact of recall bias. Third, we were unable to locate an external replication sample because of the unique nature of this cohort (e.g., very preterm neonates). The unique nature of the sample means that it is unclear to what extent our results are sample specific or whether they would generalize to later preterm or term children. Fourth, we observed differential DNAm in buccal cells rather than in the tissues that may be more clearly related to children’s health (i.e., neural tissues for neurodevelopment). However, a benefit of measuring DNAm in peripheral tissue is that it could represent processes that are occurring elsewhere in the body such as in the immune and metabolic systems. As prematurity is a systemic condition impacting nearly all organ systems, peripheral tissues may be particularly relevant to study in this sample. Finally, although the differences in DNAm we observed were small (1–6%), they are consistent with what has been reported in other epidemiological studies investigating peripheral DNAm as it relates to other prenatal risk factors (e.g., smoking [70]), as well as previous studies in our sample [37, 45]. However, small effects in DNAm are likely important [71], as they open a potential window into understanding mechanisms driving child health.

## Conclusions

In sum, we observed associations between prenatal risk factors and DNAm in very preterm infants using both cumulative risk and risk phenotype approaches. Epigenetics offers a potential biological indicator of the amount and type of prenatal risk that children were exposed to, which may be particularly useful for identifying infants at greatest risk especially in populations of vulnerable infants. There remains a need to better understand whether differences in DNAm at birth are related to children’s health and neurodevelopmental trajectories.

## Supplementary Information

**Additional file 1**. EWAS results for all suggestive associations (FDR < 10%). This Excel spreadsheet contains information about all CpGs with FDR < 10% from the models comparing physical risk to typical (Model 1), psychological risk to typical (Model 2), and cumulative risk (Model 3).

**Additional file 2**. Fit statistics for latent class analysis of prenatal risk factors. This table presents model fit statistics used to choose the optimal number of profiles in the latent class analysis.

**Additional file 3**. Differentially methylated regions (DMR) associated with prenatal risk. This table presents results from the results of the DMR analysis, including the location, estimates, *p*-values, and gene annotations associated with all significant DMRs.

## Data Availability

The raw and processed DNAm data are publicly accessible through NCBI Gene Expression Omnibus (GEO) via accession series GSE128821.

## Abbreviations

CpG: Cytosine-phosphate-guanine
DNAm: DNA methylation
DMR: Differentially methylated regions
EWAS: Epigenome-wide association study
GEE: Generalized estimating equations
GO: Gene ontology
LCA: Latent class analysis
LPA: Latent profile analysis
NOVI: Neonatal Neurobehavior and Outcomes in Very Preterm Infants
PMA: Postmenstrual age
