# Supplementary material for: Prenatal risk factors and neonatal DNA methylation in very preterm infants
Source: Clin Epigenetics. 2021 Sep 10;13:171. doi: 10.1186/s13148-021-01164-9 (PMC8434712; doi:10.1186/s13148-021-01164-9)
Supplement: Supplementary file 2 — Additional file 2. Fit statistics for latent class analysis of prenatal risk factors. This table presents model fit statistics used to choose the optimal number of profiles in the latent class analysis. [file 13148_2021_1164_MOESM2_ESM.docx]

Additional File 2

Fit statistics for latent class analysis of prenatal risk factors.

| **Classes** | **SSA BIC** | **Entropy** | **Average Class Probability** | **Smallest Class** | **LMR** | **BLRT** |
| --- | --- | --- | --- | --- | --- | --- |
| 1 | 10687.391 | - | - | - | - | - |
| 2 | 10336.218 | 0.91 | .94 - .99 | N = 157 (26%) | *p* < .001 | *p* < .001 |
| 3 | 10107.846 | 0.84 | .89 - .94 | N = 79 (13%) | *p* = .002 | *p* < .001 |
| 4 | 10083.830 | 0.76 | .82 - .96 | N = 68 (11%) | *p* = .28 | *p* < .001 |

*Note*. SSA BIC = Sample size adjusted Bayesian information criterion; LMR = Lo-Mendell-Rubin test; BLRT = Bootstrapped loglikelihood ratio test
