# Supplementary material for: Prenatal risk factors and neonatal DNA methylation in very preterm infants
Source: Clin Epigenetics. 2021 Sep 10;13:171. doi: 10.1186/s13148-021-01164-9 (PMC8434712; doi:10.1186/s13148-021-01164-9)
Supplement: Supplementary file 3 — Additional file 3. Differentially methylated regions (DMR) associated with prenatal risk. This table presents results from the results of the DMR analysis, including the location, estimates, p-values, and gene annotations associated with all significant DMRs. [file 13148_2021_1164_MOESM3_ESM.docx]

Additional File 3

Differentially methylated regions (DMR) associated with prenatal risk

| **DMR** | **CpG** | **Location** | **Est** | **P (raw)** | **P (adj)** | **Gene**  **Annotation** | **Brain-buccal correlation** |
| --- | --- | --- | --- | --- | --- | --- | --- |
| *Model 1: Physical Risk vs. Typical* | | | | | | |  |
| 1 |  | *Chr10: 133793734-133794558* |  | *4.17E-11* | *2.94E-05* |  |  |
|  | cg25123362 | Chr10:133793734 | -0.25 | 2.76E-11 |  | BNIP3 | -0.09 |
|  | cg12751948 | Chr10:133794454 | -0.05 | 3.51E-01 |  | BNIP3 | 0.19 |
|  | cg16592121 | Chr10:133794558 | -0.17 | 1.12E-04 |  | BNIP3 | -0.23 |
| *Model 1: Psychological Risk vs. Typical* | | | | | | |  |
| 1 |  | *Chr14: 77785784-77785968* |  | *4.65E-08* | *3.29E-02* |  |  |
|  | cg02181287 | Chr14: 77785784 | -0.25 | 2.83E-06 |  | GSTZ1;POMT2 | 0.00 |
|  | cg03738767 | Chr14: 77785968 | -0.23 | 9.81E-05 |  | GSTZ1;POMT2 | 0.06 |
| *Model 2: Cumulative Prenatal* | | | | | | |  |
| 1 |  | *Chr7:148843026-148844053* |  | *5.13E-10* | *3.65E-04* |  |  |
|  | cg13359301 | Chr7: 148843026 | -0.92 | 4.36E-06 |  | ZNF398 | 0.27 |
|  | cg19754614 | Chr7: 148843322 | -0.68 | 9.50E-04 |  | ZNF398 | 0.35 |
|  | cg05636131 | Chr7: 148844053 | -1.09 | 6.93E-08 |  | ZNF398 | 0.42^‡^ |
| 2 |  | *Chr14: 77785784-77785968* |  | *1.12E-08* | *7.96E-03* |  |  |
|  | cg02181287 | Chr14: 77785784 | -0.70 | 1.89E-06 |  | GSTZ1;POMT2 | 0.00 |
|  | cg03738767 | Chr14: 77785968 | -0.98 | 1.26E-05 |  | GSTZ1;POMT2 | 0.06 |
| 3 |  | *Chr3: 186965021-186965150* |  | *1.41E-08* | *1.00E-02* |  |  |
|  | cg04846432 | Chr3: 186965021 | -0.50 | 2.13E-04 |  | MASP1 | -0.01 |
|  | cg12155575 | Chr3: 186965150 | -1.16 | 8.97E-09 |  | MASP1 | 0.08 |
| 4 |  | *Chr1: 155659719-155659882* |  | *2.01E-08* | *1.43E-02* |  |  |
|  | cg06437799 | Chr1: 155659719 | -1.57 | 3.51E-06 |  | DAP3;YY1AP1 | 0.47* |
|  | cg07054239 | Chr1: 155659882 | -1.30 | 1.65E-06 |  | DAP3;YY1AP1 | -0.28 |
| 5 |  | *Chr22: 25884154-25884537* |  | *3.39E-08* | *2.41E-02* |  |  |
|  | cg07609623 | Chr22: 25884154 | -1.21 | 1.38E-04 |  | CRYBB2P1^+^ | 0.26 |
|  | cg21005828 | Chr22: 25884537 | -0.92 | 1.59E-04 |  | CRYBB2P1^+^ | -- |
| 6 |  | *Chr15: 34260712-34260956* |  | *6.47E-08* | *4.60E-02* |  |  |
|  | cg11451033 | Chr15: 34260712 | 1.49 | 1.27E-07 |  | AVEN;CHRM5 | 0.42^‡^ |
|  | cg19662219 | Chr15: 34260719 | 1.24 | 6.24E-06 |  | AVEN;CHRM5 | 0.44* |
|  | cg20369048 | Chr15: 34260872 | 1.09 | 4.46E-07 |  | AVEN;CHRM5 | 0.40^‡^ |
|  | cg26920451 | Chr15: 34260956 | 1.07 | 2.23E-06 |  | AVEN;CHRM5 | 0.31 |

Note. ^‡^*p* < .10, **p* < .05, ***p* < .01, ^+^ indicates closest gene. No data exists for one CpG (cg21005828) in the brain-buccal correlation database.
